# Parenting and pandemic pressures: Examining nuances in parent, child, and family well-being concerns during COVID-19 in a Canadian sample

**DOI:** 10.3389/fepid.2023.1073811

**Published:** 2023-04-25

**Authors:** Laura Colucci, Jackson A. Smith, Dillon T. Browne

**Affiliations:** Department of Psychology, University of Waterloo, Waterloo, Ontario, Canada

**Keywords:** COVID-19, family, well-being, parenting, child health, Canada

## Abstract

**Introduction:**

The COVID-19 pandemic has caused vast disruptions in family life for Canadian parents since early 2020. While numerous environmental stressors have been identified, including job loss and the demands of balancing work-life conflicts and at-home schooling, relatively less is known about the areas of family life parents are most concerned about and how these worries relate to well-being across the family system.

**Methods:**

Canadian parents (*n* = 29,831, 90.29% mothers, 57.40% Ontario residents) of children aged 0–14 were surveyed about their concerns related to child, parent, and family well-being in June 2020. Structural equation modelling was used to model the relationship between concerns about children, parenting, and the whole family, in association with several sociodemographic variables including child disability status, parent sex and education, job loss during COVID-19, and caregiver employment.

**Results:**

Parenting, child, and family concerns were positively correlated. Higher child and family concerns were reported by parents who had not attended university, those who had experienced employment loss or reduced hours, and families with all adults working outside the home. Parents of children with a disability reported higher concerns across all three domains: child, parenting, and family psychosocial well-being.

**Discussion:**

These results showcase distinct associations between social determinants of health and the types of worries caregivers exhibited across multiple areas of family life during the first wave of the COVID-19 pandemic in Canada. Findings are interpreted in relation to clinical intervention and public policy targets for families.

## Introduction

Parents and families have been adversely impacted by the COVID-19 pandemic (1). Seismic shifts in daily life have occurred since early 2020, with changes to work-life schedules, at-home schooling, and public health mandates (2, 3). Because of lockdowns and repeated disruptions to social and economic life, the prominence and multiplicity of stressors has led to strain across social, emotional, and occupational contexts for many Canadian families (1). However, there is still a paucity of research explicating the specific areas of family functioning parents are most concerned about, and how sociodemographic vulnerabilities relate to caregiver worries (4). There are widely used models of how adversity impacts family relationships [e.g., the family stress model, (5)]; however, far less is known about how COVID-19 has led to particular areas of parental concern (6). Most of this work has focused on caregiver “anxiety” in contexts of clinical levels of distress, thus there is a need to better understand and document the population-level, non-clinical (i.e., normative) concerns that all families are facing in order to extend working models of family stress within the pandemic context (7–10). This study sought to apply a family systems lens to the study of Canadian families during the pandemic by (a) modelling the dimensional structure of child, parent, and family well-being concerns, and (b) exploring the relation between sociodemographic factors (i.e., social determinants of health) and parents' levels of worry in different domains. This research will provide timely clarification regarding the specificity of stressors experienced by Canadian families during the first wave of the pandemic and their associations with child, parent, and family well-being.

### Parenting under pandemic pressures

Developmental science and family research is regularly focused on identifying the ways in which adversity “*gets inside the family”* (p. 398) (11). Within the family stress model framework, parents'experiences of stress and adversity (such as poverty, disaster, recession, or the pandemic) pose risks for a suboptimal environment within the family home through increased levels of couple conflict and harsh or insensitive parenting, with cascading effects on parent and child mental health and overall well-being (4, 5). Parents often experience immense pressures related to ensuring their family manages well amidst stressors and crises; thus, their relative well-being during these times may become compromised and serve to increase risks towards maladaptive patterns of adjustment (4, 12).

Increased incidence of mental health challenges has been a widespread consequence of COVID-19-related disruptions to daily life, and Canadian parents have been identified as especially vulnerable to pandemic-related stress (1). Given the myriad personal, economic, occupational, and health-related stressors many are facing during the pandemic, it is valuable to explore parents' specific perceptions of these challenges, including the factors that are associated with their variation. A recent study found evidence to demonstrate shared risks between caregiver burden, parent mental health, and deleterious impacts to the parent-child relationship during the pandemic (13). A systematic review and meta-analysis of maternal mental health in mothers of young children found high prevalence estimates of clinically significant depression (26.9%) and anxiety (41.9% overall; 36.4% after adjusting for publication bias) which were markedly increased from pre-pandemic rates [17% and 15% respectively (9)]. Further, child-specific health behaviours have also been associated with parent psychological well-being. McCormack and colleagues (7) studied self-reported anxiety levels surrounding the pandemic in Canadian parents and found that higher anxiety levels were associated with fewer health promotive physical activities and increased duration of sedentary behaviours in children. This pattern of results suggests that even subtle fluctuations in parent stress and psychological well-being are associated with widespread changes across the family unit (2).

### Vulnerabilities towards child maladjustment during COVID-19

Canadian children were identified as being less vulnerable to experiencing COVID-19 medical complications compared to older-aged adults (14). However, children have faced significant disruptions to routines and family-life, access to education, and other supports during this time, rendering them vulnerable to the onset or exacerbation of existing mental health difficulties (15). A survey of 350,000 parents in the United States found pervasive mental health challenges for children that had increased since school closures near the start of the pandemic (16). Compared to pre-pandemic levels, parents reported a greater prevalence of mental health challenges in their children (anger, anxiousness, depressed or low mood, loneliness), less positive adjustment (positive social relationships, hopefulness, outlook, overall demeanor), and less positive family interactions (sibling and whole-family dynamics), demonstrating systemic impacts from pandemic-related stressors across layers of the family system and developmental ecology (16).

Numerous studies during the pandemic have brought attention to concerns for children's academic and social development, following school closures and shifts between online and in-person schooling. According to a United Nations (17) report on global education impacts, COVID-19-associated academic losses have been considered “*the largest disruption of education systems in history”* (p. 2)*.* They estimated that almost 1.6 billion students across more than 190 countries have faced partial or complete loss of access to education during this period. In addition to educational losses, many children lost access to daycare, extracurricular activities, learning supports, and opportunities for socialization outside of the family unit (18). These disruptions were enduring and significantly negative for many children, with lost opportunities for meaning-making, socialization, and personal and academic development (19).

### Whole-family impacts of pandemic-related stressors

Though often understudied in the epidemiological literature, the family system is an emergent whole that warrants analysis as a unique and distinct entity, not merely reducible to “children and parents” (20, 21). Numerous studies have identified an ambient, family-wide relational climate in the domains of emotional positivity and negativity, sensitivity in relationships, and other interpersonal processes such as attachment and perceived closeness (22–24). For many, the family home became the hub, not only for family life, but also work, schooling, leisure, socializing, and other everyday tasks during pandemic-related closures and lockdowns. As such, families may have been susceptible to greater “spillover” of stress between domains like work and family relationships (25). Thus, applying a family-level framework when evaluating parents' COVID-19-related worries may be particularly informative for understanding how families are coping during the pandemic.

A qualitative study of families in Australia in April 2020 found that some families reported positive consequences from COVID-19 life changes (26), keeping with the concept of family resilience (27). These included greater shared workload between partners at home, increased family time and opportunities for relational connection, and a slower pace of life. Conversely, other families in the study commented on the struggles of social isolation, immunocompromised family members and worries about infection, financial burdens, balancing work, parenting challenges, and reduced (or lack of) access to psychological and physical health supports for their children.

Multinational Canadian research on family adaptation during the pandemic echo Evans et al.'s (26) findings. Based on a dataset with families from the United Kingdom, United States of America, Canada (4%), and Australia, Shoychet and colleagues (28) found several positive factors associated with family-wide benefits from COVID-19 including prioritizing family more than work, finding new meaning in life, and engaging in new family activities. Notwithstanding, another recent study of multi-level family stress and COVID-19 disruption from the same sample identified that experienced disruptions to well-being during this period were associated with differences in parenting quality and mental health status between siblings in the same family (29). From such investigations, it appears that families experienced increased stress on the one hand, and enhanced connectedness on the other. Certainly, there is widespread variability in the ways in which families were impacted by the pandemic, particularly for vulnerable groups such as single-parent families and those facing systemic barriers like poverty, racism, or marginalization (4). Further evaluation of specific factors impacting whole-family resilience and well-being will be especially valuable in understanding how to best support families as COVID-19 restrictions ease.

### The current study

This study sought to identify the domains of parents’ concerns during the COVID-19 pandemic, while exploring the sociodemographic factors that were related to those unique areas of worry. By modelling child-specific, parenting, and whole family areas of concern, this work is unique in that it offers multilevel conceptualizations of parent concerns for family well-being, while also considering social determinants of health that have been associated with increased stress and hardship. To date, much research has demonstrated the deleterious impacts of COVID-19 across the world; however, limited research has explored multiple levels within the family system and sought to isolate disparate sources of worry. This analysis was informed by two primary research questions: (1) What is the relationship amongst caregivers' concerns with parenting, children, and family? and (2) How do parent and family sociodemographic variables (e.g., economic factors, parent sex, child disability) relate to parent concerns for child-specific, parenting, and whole family well-being during the COVID-19 pandemic? Our hypotheses and analytic plans were pre-registered and can be accessed at: https://osf.io/x89cd.

## Materials and methods

### Sample

We used the *Impacts of COVID-19 on Canadians—Parenting during the Pandemic* dataset from Statistics Canada for this study, which asked parents about their parenting experiences between March-June of 2020. The *Impacts of COVID-19 on Canadians* study survey was open to participation from all adults across Canada who had a child under the age of 15 years residing in their home. Parents were recruited through online crowdsourcing (e.g., social media and outside parties like government agencies and news outlets) and data collection occurred through an anonymous Statistics Canada portal. Recruitment was initiated by Statistics Canada as the 5th iteration of crowdsourcing data collection cycles, with the goal of inviting participation from any parent in the Canadian population who met the above criterion. Participants were not randomly selected and, as such, interpretations from these data are limited to the sample that was studied and may not be reflective of all Canadian parents of a child under 15 years. The sample included *N* = 32,228 parents who participated in the survey, which asked parents about their parenting experiences between March and June of 2020. This dataset is publicly available, and Statistics Canada has previously conducted and disseminated certain analyses [e.g., (30–32)], though the research questions from this study have not been examined.

### Procedure and measures

#### Sociodemographic variables

Sociodemographic variables of interest in this study included parent sex, child disability status, and economic factors (e.g., parent education, job loss, and whether families were working inside or outside the home). In accordance with Statistics Canada's confidentiality guidelines, several sociodemographic variables were collapsed across responses to limit disclosure risk. Descriptive statistics are reported in Table 1. Regarding parent sex, parents were provided with the response options of male, female, or gender diverse. Due to limited responses, gender was benchmarked to sex and gender diverse responses were randomly assigned to either male or female by Statistics Canada for participant confidentiality. Parents in this study were categorized into four age groups by Statistics Canada: 15–34 years (19.68%), 35–44 years (64.05%), 45–54 years (15.49%), and 55+ years (0.78%).

**Table 1:** Descriptive statistics for study variables.

Variable & level		% or M	SD
Parent sex	Male	9.70	–
Child with a disability	No	83.21	–
Parent education	Did not attend university	24.14	–
Job loss or reduced hours	No	60.96	–
Employment structure	Inside home	49.12	–
	Outside home	16.36	–
	Mixed	34.52	–
Child health		1.99	0.83
Child loneliness		2.69	0.88
Child mental health		2.54	0.88
Child school/academics		2.32	1.00
Child socialization		3.04	0.82
Parent balancing		3.15	0.92
Parent managing child behaviours		2.80	0.90
Parent patience		2.54	0.94
Family connection		2.45	0.77
Family supportiveness		2.27	0.89
Family loneliness		2.07	0.98

Values reflect the number of complete cases within each level of the variable, after exclusion of missing data (i.e., “Not Stated” and “Not applicable” responses) and multivariate outliers. The range for all concern variables was 1 (Not at all concerned)-4 (Extremely concerned).

For parent education, parents were asked to report the highest level of education they had attained, ranging from “less than high school diploma or equivalent” to “University certificate, diploma, or degree above the BA level.” Responses were dichotomized by Statistics Canada into a binary variable reflecting whether parents had or had not completed university-level education. Similarly, for job loss, parents were asked to endorse yes or no to the following statement: *Someone in my family lost their job, was laid off, or has reduced work hours due to COVID-19*. To ascertain employment structure of working individuals in the home, participants were asked to endorse yes or no to the following statements: *Someone in my family is working at a fixed location outside the home; Someone in my family is working outside the home with no fixed location; Someone in my family is working from home.* Statistics Canada then collapsed the responses into 3 options for the publicly available data file: *All family members working are doing so from home*; *All family members working are doing so outside the home*; and *Mixed.* Unfortunately, no other data were collected to identify change in job role (i.e., individuals who were previously working from home prior to the pandemic).

The majority of respondents in this study were Ontario residents (57.40%), with a smaller percentage of participants from British Columbia (12.85%), Alberta (8.93%), Quebec (7.50%) and Nova Scotia (5.20%). Very few participants in this study were from other provinces and territories (<5% each from Newfoundland and Labrador, Prince Edward Island, New Brunswick, Manitoba, Saskatchewan, and the territories). Related to child age, parents were asked to report on the age brackets of children in their home (unfortunately no data was available on how many children each parent cared for). In this sample, 61.37% had a child(ren) aged 0–5 in the home and 64.14% had a child(ren) aged 6–14 in the home (see the *Missing Data* section below). Related to older teens and adult children, 8.95% of participants had a child(ren) aged 15–24, though parents were only asked to report on concerns for their children aged 0–14 in this study.

For child disability status, parents were asked whether a child under the age of 15 in their home had a disability. This included permanent physical disabilities, cognitive, behavioural, or emotional disabilities, the option to select “other disability,” or “no disability.” Responses to this question were collapsed into a dichotomous variable based on whether parents disclosed the presence or absence of any child in the home with any type of disability. Though not available in the data used in the present study, another analysis of this dataset identified that, of the parents reporting that a child in their home had a disability, 84% endorsed a cognitive, emotional, or behavioural disability, with a smaller proportion of parents reporting a permanent physical disability (4%), other disability (7%), and/or at least two types of disabilities (6%) (30). The data in this study are not able to clarify whether children had more than one type of disability and do not inform whether more than one child in the home had a disability (30). These limitations are respectively due to redacted demographic information in the publicly available dataset and the wording of the survey question related to disability, which only asked a binary (yes/no) question about the presence of *any* child in the home with a disability.

#### Concern variables

Child concerns were assessed through five items. Parents were asked, *Due to the COVID-19 pandemic, how concerned are you about the following for your child or children aged 0–14 years?* for the following domains: general physical health, general mental health, loneliness or isolation, school year and academic success, and opportunities to socialize with friends. For child concern questions, if parents had more than one child, they were asked to report the response that best captured their level of concern across all children in the home aged 0–14.

Parenting concerns were assessed through three items. Parents were asked, *Due to the COVID-19 pandemic, how concerned are you about the following for your family?* for the following domains: Balancing child-care, schooling and work; managing your child's or children's behaviours, stress levels, anxiety, emotions; and having less patience, raising your voice, scolding or yelling at your child or children. With the same starting question, parents were also asked about their degree of concern for several whole-family domains: staying connected with family or friends; getting along and supporting each other; and feeling lonely in your own home. For all concern questions, parents provided responses on a scale ranging from (1) *Not at all* to (4) *Extremely*. Parents were also able to select (5) *Not Applicable* (for child concern questions only) or skip questions. Such responses, along with missing data, were excluded from the final analysis.

#### Analysis

We conducted descriptive statistics in SPSS v. 28 (Table 1) and structural equation modelling in Rstudio version 1.2.5033 using the *lavaan* package (33, 34). We applied survey weights from Statistics Canada in the analysis of this dataset with the *lavaan.survey* package (35). When multivariate outliers were assessed with the Mahalanobis Distance Test (which uses complete cases only *n* = 29,702), 2,397 multivariate outliers were found at the *p *≤ .05 level, these were removed, leaving a sample of 29,831 from the original 32,228. The final sample size included 27,305 complete cases after removing both outliers and missing data (see section below).

**Figure 1:**
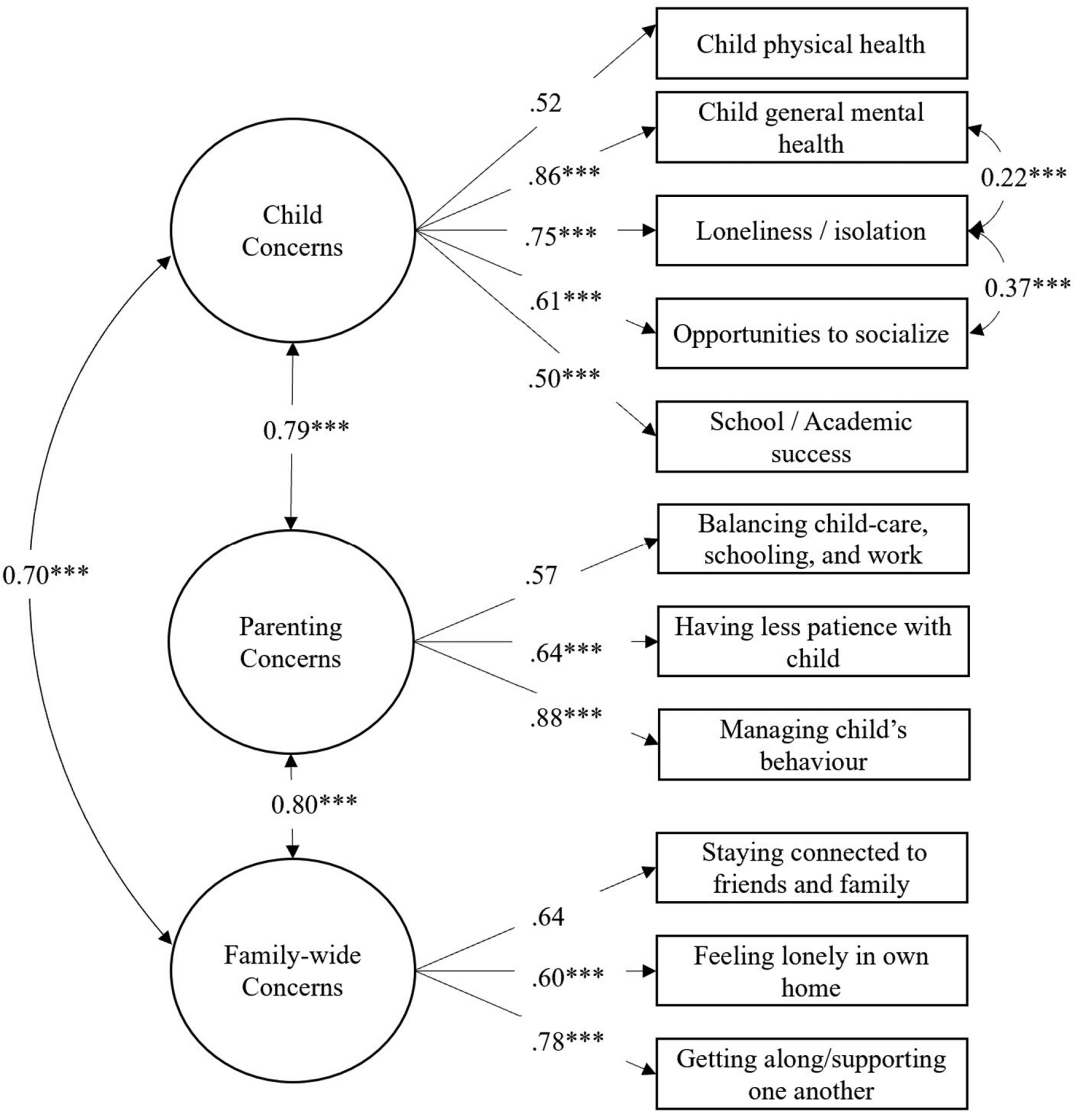
Measurement model. Circles reflect latent variables, boxes reflect factors. Double-sided arrows reflect correlations, single-sided arrows depict factor loadings. Standardized estimates reported, restricted sample depicted—without multivariate outliers. For each factor, the first indicator was fixed to 1.0. Within-factor correlations for the *Child Concerns* latent variable were added based on consideration of modification indices and theoretical justification. ***p < .001.

Confirmatory factor analysis was used to explore the hypothesized associations between parent concerns, child concerns, and family concerns. Parent responses to concern questions in the survey were respectively combined into three latent variables reflecting concerns for child(ren), parenting, and family well-being (see Figure 1). Maximum likelihood estimation with robust standard errors (MLM) was used due to restrictions with *lavaan-survey* package not correcting the standard errors in the chi-square statistic with regular maximum likelihood estimation. MLM uses the Satorra-Bentler chi-square correction statistic (36). The use of Full Information Maximum Likelihood (FIML) estimation for missing or incomplete data is not currently available with the use of survey weights in *lavaan* (37). To explore any potential differences that would have resulted from the use of this function, the measurement model (Figure 1) was tested without survey weights, using FIML (which included all cases with “missing” or “Not Applicable” responses); the fit statistics were nearly identical to the retained model. A likelihood ratio test was not able to be completed in this case due to differing sample sizes between models. When the structural model was tested with additional regressions with the full sample, a similar pattern was observed. Ultimately, for the results to be consistent with other published analyses of this dataset, we applied survey weights in the analysis without the use of FIML to handle missingness.

For the measurement model, we constructed three correlated factors (parenting concerns, child concerns, and family concerns) using the respective survey items (described above) as reflective indicators, and allowed the factors to covary. To reflect the bidirectionality and reciprocal influence between levels of the family system (consistent with existing theoretical models and empirical research) correlations between the latent variables were retained in the structural regression model (20, 24). This specification diverged from the pre-registered analytic plan to include regressions from the parent concerns to child and family concern variables, for the sake of parsimony and interpretability of results.

In the structural regression model, we regressed all three latent variables (child concerns, parenting concerns, family concerns) onto the sociodemographic variables within the model (which were dummy coded). These included: child disability status (0 = no child disability, 1 = child with a disability); parent education (0 = did not attend university, 1 = did attend university); family employment with all adults working inside the home (1), outside the home (2), or mixed employment structure (3); job loss or reduced hours (0 = did not experience, 1 = did experience), and parent sex (0 = male, 1 = female). For interpretability, after the original model was run, the family employment variable was re-coded into 3 dummy variables and multiple contrasts were run, to ascertain the differences between all three levels: inside the home compared to outside of the home, outside the home compared to mixed working arrangement, and inside the home compared to mixed working arrangement, with the first value of each variable coded as 0 and the second as 1. As such, the reference variable changed between contrasts. The following specifications were utilized to evaluate model fit: a non-significant chi-square test, Comparative Fit Index (CFI) ≥ .95, Root Mean Square Error of Approximation (RMSEA) < .06, and Square Root Mean Residual (SRMR) < .08 (38).

### Missing data

The dataset contained few missing responses (between 22 and 52 cases, <1%) across all concern variables due to skipped questions. Similarly, across all but one of the five child concern variables (concern for children's school/academic success), a very small number of participants selected “Not Applicable” for their responses (between 87 and 103 cases). Conversely, there were 2120 cases (7.12%) where parents reported “Not Applicable” for concerns related to children's school/academic success, resulting in missing data from the dataset. Of parents who reported “Not applicable” for concerns related to children's academic success, 99.48% (*n* = 2,109) were parents who also reported having a child(ren) aged 0–5 years old in the home. Similarly in this subgroup, a very small proportion of respondents reported having a child(ren) aged 6–14 (*n* = 16, 0.7%), suggesting that these responses were selected by parents of infants or young children that were not yet attending school. To explore how the absence of data related to the child concerns variable impacted the overall distribution of parents of children of varying ages, we ran separate frequencies to assess child ages with this exclusion specifically; however, the final analysis excluded any case with missing data across any variable. Thus, the final sample is approximately composed of parents of a child(ren) aged 0–5 years (40.8%), 6–14 years (72.7%), and 15–24 years (11.4%)**.**

## Results

When the original measurement model was fit and tested, it achieved adequate model fit (*n* = 27,305; *χ*^2^(41) = 6,333.36, *p* < .001, CFI = .92, RMSEA = .094 (CI = .092–.095), SRMR = .041). All three latent variables were significantly positively correlated (Child and parent concerns: *r* = .76; child and family concerns: *r* = .69, parent and family concerns: *r* = .80; *p*s < .001) and all the specified factor loadings for each latent variable were statistically significant (*p*s < .001; Figure 1). Modification indices suggested adding within-factor correlations between several indicators: (1) between concerns about child loneliness and child opportunities to socialize, and (2) between concerns about child loneliness and child mental health. Given that these were theoretically justified, these two correlations were added to the model, which significantly improved model fit [Likelihood ratio test: *χ*^2^(2) = 1,383.7, *p* < .001, note, this test compares the *non-adjusted* chi-square statistic between both models]. Fit indices for the adjusted model were as follows: n = 27,305; *χ*^2^(39) = 4,696.19, *p* < .001, CFI = .94, RMSEA = .082 (CI = .080–.084), SRMR = .040. The correlation matrix for the data can be found in the Supplementary Material accompanying this article. Similarly, when the structural model was tested with the addition of regressions for the sociodemographic variables, the model fit was within the acceptable range, though the CFI was slightly lower than the recommended ≥.95 cut off: *n* = 20,244; *χ*^2^(79) = 4460.84, *p* < .001; CFI = .924; RMSEA = .065 (CI = .063–.067); SRMR = .034 (38).

Several significant associations were found when the path model was tested (see Table 2 for path estimates). As in the measurement model, parenting concerns were positively correlated with family (*r* = .80) and child concerns (*r *= .77), and family and child concerns were also positively correlated (*r* = .68, *p*s < .001). No significant parent sex differences were found for any of the parent concern variables. Though parents demonstrated commensurate *degrees* of concern for child, parenting, and family-based factors, additional exploratory analyses were conducted to identify patterns in the sociodemographic *predictors* of concerns between male and female caregivers by re-running the analyses in sex-separated subsets of the data (see the accompanying Supplementary Material). When these analyses were conducted, many of the associations were non-significant for male caregivers. Other associations were maintained from the original model but were reduced in magnitude (e.g., Child disability predicting higher child and parenting concerns, job loss predicting higher family concerns, and mixed employment structure being associated with higher child concerns than when all adults were working either inside or outside of the home).

**Table 2:** Structural regression model parameter estimates for social determinants of health in association with parent-reported concerns.

Variable	Child concerns		Parenting concerns		Family concerns	
	Unstandardized (SE)	Standardized	Unstandardized (SE)	Standardized	Unstandardized (SE)	Standardized
Male (vs. Female)	0.01 (0.12)	0.00	0.03 (0.02)	0.02	−0.01 (0.02)	−0.01
Completed university	**−0.06** (**0.01)**	**−0**.07***	0.01 (0.01)	0.00	**−0.05** (**0.01)**	**−0**.04***
Job/hours loss	**0.03** (**0.01)**	**0**.04***	0.01 (0.01)	0.01	**0.07** (**0.01)**	**0**.7***
Child disability	**0.16** (**0.01)**	**0**.17***	**0.20** (**0.01)**	**0**.15***	**0.08** (**0.01)**	**0**.07***
Employment	** **		** **		** **	
In vs. out	**0.03** (**0.01)**	**0**.04**	**−0.07** (**0.02)**	**−0**.06***	**0.05** (**0.01)**	**0**.4***
In vs. mixed	**0.03** (**0.01)**	**0**.04***	0.01 (0.01)	0.01	0.01 (0.01)	0.01
Out vs. mixed	0.00 (0.01)	0.00	**0.09** (**0.02)**	**0**.08***	**−0.03** (**0.02)**	**−0**.03*

***Note:*** Variables were dummy coded such that **males**, parents who **did not** complete university, parents who **did not** experience job loss and those **without** a child with a disability were all coded 0.

Significant paths bolded: *p ≤ .05, **p ≤ .01, ***p ≤ .001.

Specific to economic factors, parents who had not attended university and parents who had experienced job loss or reduced hours during the pandemic reported greater family and child concerns in this sample, with non-significant differences for parenting concerns. Regarding child disability status, parents who had a child under 15 with a disability reported more concerns across all three variables, with significantly greater child, parenting, and family concerns than parents without a child with a disability in the home.

Subsequently, this model was re-run in three iterations to test the differences between all 3 levels of the employment structure variable with dummy coding [0 = inside vs. 1 = outside the home (*n* = 14,087); 0 = inside vs. 1 = mixed employment structure (*n* = 16,785); and 0 = outside the home vs., 1 = mixed employment structure (*n* = 9,616)]. Parents of families with all adults working outside of the home reported greater concerns for family and child well-being compared to families where all adults were working inside the home. They also reported more family concerns than parents in a mixed employment structure. Conversely, parents with all adults working inside the home, or in a mixed employment structure, reported a greater number of parenting concerns than families with all adults working outside the home. Lastly, among families where all adults wereworking inside the home, parents reported greater concerns for child well-being when there was a mixed employment structure, with non-significant differences for parenting and family concerns.

## Discussion

This study explored Canadian parents' concerns related to multiple aspects of family well-being during the first wave of the COVID-19 pandemic. In this study, parent worries were clustered in the spheres of child, parenting, and whole-family well-being and were positively associated with one another. These findings align with other family studies that have explored interpersonal processes within and between levels of family functioning (23, 24). They also support bidirectional relational processes across levels of the family, demonstrating an interplay or “spillover” between parent concerns in one domain and related changes across the family system (39). The results of this analysis support systems conceptualizations of family functioning and clarify unique patterns of whole-family disruption related to sociodemographic factors (12, 20). Our findings depict unique manifestations of parent-concerns, differentially occurring at various levels of the family system, and demonstrate the value of modelling pandemic disruption from a family systems lens.

### Sociodemographic stressors on the family unit

This study found several sociodemographic stressors to be associated with parent-reported concerns for well-being across the family system, supporting the tenets of the family stress model framework (12). Our findings also support the putative pathways within the family stress model, which posits increased risk for maladjustment (in this case, degree of parental concerns) in the context of socio-economic pressures—a prominent concern for many families during this time (5, 6). The observed interplay between parent-reported concerns and sociodemographic stressors across spheres of family functioning is critical given that parents were experiencing increased stressors due to difficulty with work-life balance during the pandemic and many children suffered academic, social, and personal losses (4). This suggests that the proliferation of pandemic-related stressors may “get inside the family” through interpersonal exchanges across the family system and may also have a direct impact on parent perceptions of child mental and physical health (29). These results also highlight potential pathways of resilience in the quality of parent-child interactions, aligning with research suggesting that nurturant and involved parenting supports positive child adaptation, even during times of stress and economic risk (4, 40).

#### Economic factors predicting child and family concerns

This study evaluated several economic factors in relation to parent-reported concerns, including parent education, job loss or reduced hours during the pandemic, and home employment structure. Related to parent education, parents who had not attended university reported a higher degree of family-wide and child well-being concerns. This finding contradicts those of Vogelbacher and Attig (41) who found that higher educated parents reported more stress. However, parents who had not attended university may have been facing greater financial concern during the first wave of the pandemic, which could have led to strain on family relationships and concern for child well-being (though this may not characterize all parents). Parallel findings were observed for job loss or reduced hours at work due to COVID-19 in this study, with affected parents reporting greater family and child well-being concerns than those who had not experienced occupational disruption. Parents in these circumstances were likely to be spending more time at home with fewer role conflicts between work and home(which may underscore the non-significant relationship to parenting concerns); however, they may have also been job-seeking, caring for children or family members, or experiencing non-work-related stressors that could have enhanced the ambient level of stress in the home, limited positive benefits related to increased time for family-bonding, or restricted financial resources for families (25).

Unique differences in family concerns were observed based on family employment structure in this study. Families that had all adults working inside the home or a mixed employment structure reported a higher degree of parenting concerns related to caring for children compared to families with all adults working outside the home. Parents working from home may have experienced both the benefits of at-home presence with children and lower coronavirus infection risk, leading to fewer concerns for child health and family interpersonal dynamics, but also the challenges of juggling the balance of work and childcare, the task of supporting their child(ren) through virtual learning, and/or challenges with a cramped living and working space. Conversely, the greater number of child and whole-family concerns reported by parents working outside compared to inside the home may reflect heightened home-stress or family chaos for parents whose jobs were not hindered in the same way by the lockdown measures (e.g., healthcare workers or those working in essential service sectors that were stressed beyond regular capacity during the pandemic). Parents in these work sectors may have also specifically faced challenges with restricted freedom to take leaves from work (42). As a result, parents working outside the home may have experienced heightened concerns for their own, their children's, and their family's health due to concerns about COVID-19 exposure and loss of opportunities to remain at home with children during this period (43). These parents may have had limited access to daycares and child supervision, which also may have increased concerns about general psychological well-being and opportunities for early-learning and socialization (43). Collectively, these results align with other studies during COVID-19 highlighting that the overall degree of economic risk posed towards families is associated with disruptions in well-being (44).

Our results showed commensurate levels of concern across mothers and fathers in this sample, related to parenting, child, and family well-being. This similarity is consistent with an analysis of family adaptation during COVID-19 by Shoychet and colleagues (28) who observed that the factor structure of perceived family coping and adaptation during the pandemic was similar across caregiver sex. Results from exploratory analyses demonstrated some, though comparatively fewer significant associations between socioeconomic predictors and the degree of paternal concerns when the statistical model was independently tested in sex-stratified analyses. Overall, this result suggests that female and male caregivers are similarly concerned for child, parenting, and family well-being (a commensurate *degree* of concerns was reported across groups in the same model), and that the *predictors* of those concerns may vary, though the difference in the magnitude of these associations between male and female caregivers was not explicitly measured (i.e., Differences in the statistical significance of parameters between models do not necessarily inform whether those parameters significantly vary from one another). Together, these findings demonstrate the value of considering both maternal and paternal perspectives in family well-being research (45, 46).

#### Increased need and loss of supports: Parent concerns for children with disabilities

Families of children with a disability were disproportionately affected during COVID-19 due to lack of access to specific services (30). This study found that parents of a child(ren) with a disability reported a greater degree of family, parenting-related, and child well-being concerns than the general population of parents. In another analysis of this dataset focused on Canadian children with disabilities, parents of children with disabilities were significantly more likely to report concerns specifically related to managing children's behaviours and emotional well-being, school year and academic success, and mental health compared to parents of children without disabilities (30). One study on the impact of COVID-19 on families of children with autism found that families more intensely experienced challenges that were present pre-pandemic during the early lockdown periods [aligned with the timeline of data collection in this study (47)]. For many families, pandemic-related closures and lockdowns meant that they experienced disruptions to everyday routines, difficulties managing parent work schedules alongside childcare, delays in receiving assessment or treatment services, and their children requiring more one-on-one support in the absence of school-based and other specialized services (26, 47, 48). Putting these results in context, parents of children with disabilities reported greater concerns across all family domains–both proximal day-to-day challenges during the pandemic surrounding children's academics, time management, or overall psychosocial well-being, and distal family-related concerns regarding overall interpersonal dynamics and relational well-being within the broader family ecology.

#### Parental resilience to pandemic pressures

Surprisingly, few of the included sociodemographic stressors were associated with parenting concerns (other than child disability and family employment structure). This pattern overlaps with findings from a longitudinal study evaluating family functioning pre-post pandemic in the United States, suggesting substantial impacts of COVID-19 disruption on child internalizing and externalizing problems and parent depression, but comparatively smaller effects for (co)parenting quality (44). Furthermore, our findings of whole family differences in the absence of detriments to the parent-child relationship also parallel an Australian study of families pre- and post-pandemic between March 2020 and August 2021. Overall and colleagues (49) demonstrated declines in parent psychological and physical health and couple and family functioning across this period (e.g., higher problem severity and family chaos, and less family commitment and cohesion), but found no differences in parent-child relationship quality or parenting practices between lockdowns. It is possible that parent-child relationships were an area of resilience among parents in this sample, buffering them from the risks associated with pandemic-related economic losses (4). Amidst the stressors of the initial lockdowns, parent-child interpersonal dynamics may have been one component of the pandemic over which parents had relatively more control–maintaining sensitivity towards their child(ren)'s needs, enhanced caregiving due to fewer time-demands outside the family home, and increased opportunities for relational connection (26).

More broadly, considering the factor structure of the parent concern variable, the results of this analysis capture child-directed parenting concerns related to balance of schedules and responsiveness to child(ren); it is possible that parents were more concerned about other factors that were not captured in the dataset (and therefore, the analysis) such as pandemic-related food and resource accessibility, the well-being of extended family and friends, or their own mental or physical health [as discussed by Fisher and colleagues (6)]. Furthermore, parenting concerns appeared most prominent for adults at home and those in mixed employment structures. Our findings align with studies demonstrating that parenting challenges are greater in the presence of role-conflicts and when there are fewer coparenting supports (25, 50). They may also be reflective of individual factors such as coping style and personality, which were not measured in this sample but were observed to predict worry surrounding lockdowns in an Italian sample during the first wave of the pandemic (51).

### Limitations and future directions

A primary limitation of this analysis is its cross-sectional design, though our results align with other longitudinal studies conducted in the early waves of the pandemic [e.g., (46)]. Future studies should continue to evaluate the long-term family-wide sequalae that emerge as the response to COVID-19 evolves and families adapt to life with fluctuating pandemic restrictions. Additionally, the dataset used in the present study lacks information pertaining to parent mental health, and child concerns were averaged across all children in the family, precluding analyses of sibling differences and parent psychological well-being. Future studies should consider these factors and continue to apply systems-level conceptualizations of family stress when evaluating family well-being [see (1, 29)]. Due to the sampling design, the results are not necessarily representative of all Canadian parents of a child under 15 and are limited to those included in this sample. Relatedly, though this study included both male and female caregivers, the latter were over-represented. Lastly, due to missing data surrounding school attendance in very young children, our findings may not generalize to single-child households of parents with a child aged 0–5 years.

#### Relevant intervention and public policy targets

Concerns for well-being were an unfortunately normative experience for many parents during the pandemic. Notwithstanding, parent and child mental health are not isolated to individuals but occur within the interpersonal climate of the whole family system (2, 25). Clinical implications of this work include the application of a family-wide framework to clinical service provision across broad healthcare spheres. These data support that care-providers—particularly for children and families—should be aware of both the family stress and family systems frameworks when considering post-pandemic parenting concerns (20, 40). Our results are complimentary to the findings from a longitudinal study during the pandemic by Calvano and colleagues (52) that advocate for family-oriented intervention efforts to mitigate risks for both parents and children in relation to parent-reported stress and psychological well-being during the course of the pandemic. Thus, it is critical that risk-mitigating policy targets are also considered in view of the results from the current study. Several recommendations are proposed.

1. On-going availability of government funding for those affected by job-loss or reduced employment hours due to pandemic-related closures. The results of this study—demonstrating associations between job loss and enhanced parent concerns for child and family—are aligned with other studies of pandemic-related child and family stress, and the broader family stress model framework that links economic stress and disruptions to family well-being (5, 52). The Canadian Government responded to the COVID-19 crisis with numerous financial support offerings for those affected by job and income loss due to pandemic-related factors. These supports should continue to be available as Canadian citizens face the current post-lockdown recovery period.
2. Enhanced social and financial supports for families with children who have disabilities. These results demonstrate that parents in this group may be particularly vulnerable to enhanced child, parenting, and family well-being concerns during COVID-19 due to loss of external supports and reduced access to services [e.g., in-person schooling and learning supports, medical, and psychosocial assessment and intervention services, alongside loss of child-care—specific challenges that have been highlighted by this population to date (26, 47)].
3. Increased availability and accessibility of mental health services for parents and families. Though parent mental health was not directly measured in this study, our results demonstrate that sociodemographic factors and COVID-19 stressors were significantly related to the degree of concern parents reported for their family's well-being (including child mental health). Previous studies have identified high prevalence rates of parent and child mental health challenges during the pandemic (1) and these findings demonstrate that parent concern during this time spanned multiple domains of well-being in the family. Thus, increased government funding should be allocated to expanding public access to mental health care for parents and children, particularly for families facing barriers to service access [echoing the recommendations of Racine and colleagues (53) who discuss the limitations of COVID-19-related changes in mental health interventions].

### Conclusion

This work is novel as it models multiple levels of family organization, analyzing whole-family well-being from a systems framework. Our findings advance existing research on the pertinence of family-wide analysis in the developmental context and highlight areas of family-need in response to the COVID-19 pandemic (4, 11). Though some studies have explored well-being at more than one level of the family system [e.g., (44)], this study provides nuance and specificity related to areas of parental concern, in addition to the role of various social determinants of health. Using a large Canadian sample (*n* = 27,305), unique insights into the family system emerged: economic risk factors appeared to inform parental worries most prominently for the whole family and child(ren), while fewer parenting concerns were found, demonstrating resilience within the parent-child relationship. These results inform putative pandemic-related pathways of risk for families, reflected in parent worries for children, parenting, and whole-family well-being. Findings demonstrate the value of modelling the bidirectionality and multi-level nature of the family system during and beyond the COVID-19 crisis, depicting points of vulnerability for families during the first wave of this global crisis.

## Data Availability

The datasets presented in this study can be found in online repositories. The names of the repository/repositories and accession number(s) can be found below: This data was accessed through the Southwestern Ontario Research Data Centre at the University of Waterloo, accessible at the following link: https://uwaterloo.ca/southwestern-ontario-research-data-centre/. This enabled access to the Crowdsourcing: Impacts of COVID-19 on Canadians survey data from Statistics Canada through the Odesi platform, accessible at the following link: http://odesi2.scholarsportal.info/webview/.
