# Supplementary material for: Parenting and pandemic pressures: Examining nuances in parent, child, and family well-being concerns during COVID-19 in a Canadian sample
Source: Front Epidemiol. 2023 Apr 25;3:1073811. doi: 10.3389/fepid.2023.1073811 (PMC10910915; doi:10.3389/fepid.2023.1073811)
Supplement: Supplementary file 1 [file Datasheet1.pdf]

Parenting and Pandemic Pressures: Examining nuances in Parent, Child, and Family Well-being Concerns during COVID-19 in a Canadian Sample

Colucci, L, Smith, J.A., Browne, D.T

University of Waterloo

Supplemental Material

Tables

Table 1

Correlation Matrix for Included Variables

	Concern for:	Child Health	Child Loneliness	Child Mental Health	Child School / Academics	Child Socialization	Parent Balancing	Parent Managing Behaviours	Parent Patience with Child	Family Connection	Family Supportiveness	Family Loneliness
1.	Child Health	-										
	Child Loneliness	.36**	-									
	Child Mental Health	.47**	.71**	-								
	Child School / Academics	.27**	.35**	.4**	-							
	Child Socialization	.21**	.63**	.5**	.33**	-						
2.	Parent Balancing	.16**	.31**	.32**	.26**	.31**	-					
	Parent Managing Behaviors	.30**	.55**	.62**	.37**	.44**	.49**	-				
	Parent Patience with Child	.16**	.35**	.37**	.23**	.32**	.4**	.55**	-			
3.	Family Connection	.26**	.39**	.36**	.23**	.4**	.28**	.37**	.27**	-		
	Family Supportiveness	.29**	.37**	.42**	.27**	.30**	.35**	.52**	.46**	.53**	-	
	Family Loneliness	.24**	.41**	.37**	.24**	.33**	.22**	.41**	.44**	.37**	.45**	-

Note: Concern variables respectively reflect indicators for the 1. Child Concern, 2. Parent Concern, and 3. Family Concern latent variables
**Correlation is significant at the $p < 0.01$ level (2-tailed). Listwise case deletion for incomplete cases, chart includes all data from the restricted sample (without multivariate outliers).

Additional Exploratory Analysis for Female and Male Caregivers

Descriptive information

Descriptive statistics for the female and male caregiver subsets of the data are included in Table 2. The sample of male caregivers in this dataset included n = 2895 participants. Missing data across this subset of the data was as follows: Child with a disability (2.69%), parent education (2.66%), job loss or reduced hours (16.72%), employment structure (20.45%), and all concern variables (<0.1-0.5% for all variables except parent concerns about child school/academics (6.67%)). The sample of female caregivers in this dataset included n = 26936 participants. Missing data across this subset of the data was as follows: Child with a disability (0.50%), parent education (0.27%), job loss (15.46%), employment structure (18.47%), and all "concern" (Child, parent and family) variables (<0.1-0.5% for all variables except parent concerns about child school/academics (7.28%)).

Analysis

The same confirmatory factor analysis procedure was applied to the respective mother and father data subsets.

Female Caregivers Only

The final measurement model from the manuscript (which includes four within-factor correlations) was tested and the model fit was within the acceptable range, though the CFI was slightly lower than the recommended ≥.95 cut off (Hu & Bentler, 1999). Model fit was as follows: n = 24649 $\chi^2(39) = 4296.13$, *p* <. 001, CFI = .939, RMSEA = 0.082 (0.080-0.084), SRMR = .044. All three latent variables were significantly positively correlated (Child and parent concerns: *r* = .77; child and family concerns: *r* = .68, parent and family concerns: *r* = .79; *p*s < .001) and all the specified factor loadings for each latent variable were statistically significant (*p*s < .001). Similarly, when the structural model was tested with the addition of regressions for the sociodemographic variables, the model fit the data well, though the CFI was slightly lower than the recommended ≥.95 cut off. Model fit: n = 18368**;** $\chi^2(71) = 4132.56$, *p* <.001; CFI = .923; RMSEA = .069 (CI = .067-.071); SRMR = .037. Path estimates are depicted in Table 3.

Male Caregivers Only

The final measurement model from the manuscript (which includes two within-factor correlations) was tested and achieved was within the acceptable range, though the CFI was slightly lower than the recommended ≥.95 cut off (Hu & Bentler, 1999). Model fit was as follows: n = 2656, $\chi^2(39) = 444.74$, *p* <. 001, CFI = .944, RMSEA = 0.079 (0.073-0.086), SRMR = .038. All three latent variables were significantly positively correlated (Child and parent concerns: *r* = .82; child and family concerns: *r* = .71, parent and family concerns: *r* = .84; *p*s < .001) and all the specified factor loadings for each latent variable were statistically significant (*p*s < .001). Similarly, when the structural model was tested with the addition of regressions for the sociodemographic variables, the model fit the data well, though the CFI was slightly lower than the recommended ≥.95 cut off. Model fit: n = 1876**;** $\chi^2(71) = 406.72$, *p* <.001; CFI = .935; RMSEA = .064 (CI = .058-.070); SRMR = .034. Path estimates are depicted in Table 3.

Table 2

Descriptive Statistics for Study Variables stratified by Parent Sex

Variable & Level		Male		Female	
		% or M	***SD***	***% or M***	***SD***
Parent Age (Years)	*15-34*	14.02	--	20.29	--
	35-44	62.69	--	64.20	--
	45-54	21.13	--	14.88	--
	55+	2.14	--	0.63	--
			--		--
Child with a Disability	*No*	88.29	--	82.68	--
Parent Education	*Did not attend University*	18.79	--	24.65	--
Job Loss or Reduced Hours	*No*	67.27	--	60.30	--
Employment Structure	*Inside home*	61.88	--	47.79	--
	Outside home	7.86	--	17.25	--
	Mixed	30.26	--	34.96	--
Child Health		2.00	0.85	2.00	0.842
Child Loneliness		2.50	0.88	2.71	0.89
Child Mental Health		2.64	0.89	2.56	0.89
Child School / Academics		2.47	1.17	2.52	1.18
Child Socialization		3.06	0.81	3.05	0.82
Parent Balancing		3.15	0.89	3.15	0.92
Parent Managing Child Behaviours		2.75	0.90	2.81	0.91
Parent Patience		2.49	0.95	2.55	0.94
Family Connection		2.43	0.76	2.45	0.77
Family Supportiveness		2.28	0.88	2.26	0.89
Family Loneliness		2.04	0.94	2.08	0.98

Note: Values reflect the number of complete cases within each level of the variable, after exclusion of missing data (i.e., “Not Stated” and “Not applicable” responses) and multivariate outliers. The range for all concern variables was 1 (Not at all concerned)-4 (Extremely concerned).

Table 3

Structural regression model parameter estimates for social determinants of health in association with parent-reported concerns, by parent sex

Parent Sex	Variable	Child Concerns		Parenting Concerns		Family Concerns	
		Unstandardized (SE)	Standardized	Unstandardized (SE)	Standardized	Unstandardized (SE)	Standardized
Female	Completed University	**-0.06 (0.01)**	**-0.07*** **	0.01 (0.01)	0.01	**-0.05 (0.01)**	**-0.04*** **
	Job/hours Loss	**0.03 (0.01)**	**0.04*** **	0.01 (0.01)	0.01	**0.07 (0.01)**	**0.7*** **
	Child Disability	**0.16 (0.01)**	**0.17*** **	**0.21 (0.01)**	**0.16*** **	**0.09 (0.01)**	**0.07*** **
	Employment						
	• *In vs Out*	**0.03 (0.01)**	**0.04** **	**-0.06 (0.02)**	**-0.06*** **	**0.05 (0.01)**	**0.5*** **
	• *In vs Mixed*	**0.02 (0.01)**	**0.03** **	0.01 (0.01)	0.01	0.01 (0.01)	0.01
	• *Out vs Mixed*	-0.00 (0.01)	-0.00	**0.08 (0.02)**	**0.07*** **	**-0.04 (0.02)**	**-0.04* **
	**Variable**	**Child Concerns**		**Parenting Concerns**		**Family Concerns**	
		Unstandardized (SE)	Standardized	Unstandardized (SE)	Standardized	Unstandardized (SE)	Standardized
Male	Completed University	-0.04 (0.04)	-0.04	-0.02 (0.05)	-0.01	-0.07 (0.04)	-0.06
	Job/hours Loss	0.02 (0.03)	0.02	0.02 (0.04)	0.01	**0.08 (0.03)**	**0.8* **
	Child Disability	**0.12 (0.04)**	**0.10** **	**0.14 (0.06)**	**0.08* **	0.03 (0.05)	0.02
	Employment						
	• *In vs Out*	0.05 (0.05)	0.04	-0.13 (0.08)	-0.07	0.03 (0.06)	0.02
	• *In vs Mixed*	**0.07 (0.03)**	**0.07*~**	0.03 (0.04)	0.02	0.05 (0.04)	0.05
	• *Out vs Mixed*	0.03 (0.01)	0.02	**0.17 (0.08)**	**0.12* **	0.07 (0.07)	0.05

Note: All variables were dummy coded such that parents who **did not** complete university, parents who **did not** experience job loss and those **without** a child with a disability were all coded 0. Significant paths bolded: *~p = .047, *$p \leq .05$, **$p \leq .01$, ***$p \leq .001$
